# Beneficial Effects of *Trillium govanianum* Rhizomes in Pain and Inflammation

**DOI:** 10.3390/molecules21081095

**Published:** 2016-08-20

**Authors:** Shafiq Ur Rahman, Achyut Adhikari, Muhammad Ismail, Muhammad Raza Shah, Muhammad Khurram, Muhammad Shahid, Farman Ali, Abdul Haseeb, Fazal Akbar, Marcello Iriti

**Affiliations:** 1 Department of Pharmacy, Shaheed Benazir Bhutto University, Sheringal, Dir (U)-18000, Pakistan; pharmacistkhurram@hotmail.com; 2 H. E. J. Research Institute of Chemistry, International Center for Chemical and Biological Sciences, University of Karachi, Karachi 75270, Pakistan; adhikarimine@yahoo.com (A.A.); raza_shah01@yahoo.com (M.R.S.); 3 Department of Pharmacy, University of Peshawar, Peshawar 25120, Pakistan; m_ismail@upesh.edu.pk (M.I.); shahidsalim_2002@hotmail.com (M.S.); 4 Department of Chemistry, Shaheed Benazir Bhutto University, Sheringal, Dir (U)-18000, Pakistan; alinuml@gmail.com; 5 Department of Clinical Pharmacy, College of Pharmacy, Umm Al Qura University, Makkah 21514, Saudi Arabia; amhaseeb@uqu.edu.sa; 6 Center for Biotechnology and Microbiology, University of Swat, Swat 19200, Pakistan; fazalakbar@uswat.edu.pk; 7 Department of Agricultural and Environmental Sciences, Milan State University, Milan 20133, Italy

**Keywords:** Trilliaceae, anti-inflammatory, analgesic, oxidative burst, pennogenin, borassoside E

## Abstract

*Trillium govanianum* rhizome is used as an analgesic and anti-inflammatory remedy in traditional medicine in northern Pakistan. In an attempt to establish its medicinal value, the present research evaluated the analgesic and anti-inflammatory potential of *T. govanianum*. The in vivo anti-inflammatory activity of extract and fractions was investigated in the carrageenan induced paw edema assay. The in vitro suppression of oxidative burst of extract, fractions and isolated compounds was assessed through luminol-enhanced chemiluminescence assay. The in vivo analgesic activity was assayed in chemical and thermal induced nociceptive pain models. The crude methanol extract and its solvent fractions showed anti-inflammatory and analgesic responses, exhibited by significant amelioration of paw edema and relieve of the tonic visceral chemical and acute phasic thermal nociception. In the oxidative burst assay, based on IC_50_, the crude methanol extract and *n*-butanol soluble fraction produced a significant inhibition, followed by chloroform and hexane soluble fractions as compared to ibuprofen. Similarly, the isolated compounds pennogenin and borassoside E exhibited significant level of oxidative burst suppressive activity. The in vivo anti-inflammatory and analgesic activities as well as the in vitro inhibition of oxidative burst validated the traditional use of *T. govanianum* rhizomes as a phytotherapeutic remedy for both inflammatory conditions and pain. The observed activities might be attributed to the presence of steroids and steroid-based compounds. Therefore, the rhizomes of this plant species could serve as potential novel source of compounds effective for alleviating pain and inflammation.

## 1. Introduction

It is highly desired to discover excellent remedies for diseases that are economical, having no or low side effects, potent and efficacious in various pathological conditions. For discovering such products, medicinal plants and herbal medicines can be the best choice as plants produce a wide range of bioactive compounds, making them a rich source of different types of medicines [1]. Numerous drugs have entered international pharmacopoeias through ethnopharmacological and traditional medicine studies [2]. Based on folkloric use, research on medicinal plants has proved the presence of valuable pharmacologically active compounds with anti-cancer, antiparasitic, antifungal, antibacterial, analgesics, and anti-inflammatory properties [3,4,5]. In developing countries, majority of population depends largely on traditional system of medicines because of their low cost and ease of availability [6].

At present, available drugs for the management of pain and inflammatory conditions are either narcotic, non-narcotic analgesics or corticosteroids, possessing well known side and adverse effects [7]. Furthermore, synthetic drugs are very expensive to develop since, for the successful introduction of a new drug product hundreds and thousands of compounds are to be synthesized, screened and tested which involves a huge expense as well as time. On the other side many medicines of plant origin have been used since long with minimum or none side and adverse effects [8]. It is, therefore, inevitable to search medicinal plants for effective analgesic and anti-inflammatory molecules with significant therapeutic potentials.

*Trillium govanianum* belongs to the genus *Trillium* (family: Melanthiaceae *alt*. Trilliaceae) is an indigenous medicinal plant of Pakistan, found at an altitudinal range of 2500–3800 m [9]. In traditional medicines, rhizomes of this plant species are used for treating wounds, dysentery, skin boils, infections, and menstrual and sexual disorders [10,11,12]. The genus *Trillium* consists of 31 species, widely distributed from the western Himalayas to Japan, China, Kamchatka (Russia) and North America [13] and is an important source of bioactive compounds of different classes like steroids, glycosides, terpenoids, sterols, saponins, sapogenins and flavonoids [14,15,16]. Literature studies indicate that plant species of this genus have been extensively used as remedy for various diseases like *T. tschonoskii* has been traditionally used in China, for at least one thousand years, for treatment of neurasthenia, giddiness, headache, removing carbuncles and ameliorating pains [17,18,19]. The marked inhibitory activity against COX-2 production in macrophagocytes of the mouse abdominal cavity by isolated compounds from *T. tschonoskii* has also been reported [20]. It has also been described that the ethanol, ethyl acetate and *n*-butanol extracts of *T. tschonoskii* significantly suppress carrageenan-induced edema in rats [21]. The rhizomes of *T. erectum* called ‘beth root’ have been used in folk medicine for the treatment of hemorrhages of uterus, urinary tract and lungs [22].

Phytochemical analyses of *T. govanianum* rhizomes have resulted in isolation of steroids and saponins from the chloroform and *n*-butanol soluble fractions [23]. Moreover, antifungal and anticancer activities in addition to ethnomedicinal relevance of *T. govanianum* have also been recently reported [24,25]. However, to best of our knowledge studies concerning the therapeutic efficacy of *T. govanianum* as an analgesic and anti-inflammatory agent have not been carried out. Therefore, in present study, we evaluated the crude methanol extract and solvent soluble fractions thereof for analgesic and anti-inflammatory activities, using in vivo animal models, while the isolated compounds in our previous study [23] were tested for inhibition of oxidative burst using luminol-enhanced chemiluminescence assay.

## 2. Results and Discussion

### 2.1. Acute Toxicity

#### 2.1.1. In Vivo Toxicity Test

No overt behavioral change was reported during an observation time of 2 h post crude methanol extract administration. As shown in Table 1, maximum mortality was observed at a dose of 6000 mg/kg while no lethality was observed at 500 mg/kg dose. From the LD_50_ value (2030.4 mg/kg), the crude methanolic extract is considered as safe to the maximum of tested dose used in this study.

#### 2.1.2. In Vitro Toxicity Test

The crude methanolic extract and its fractions were tested in vitro against NIH 3T3 mouse embryo fibroblast cell line to ascertain their cytotoxicity. As shown in Table 2, the IC50 values for the in vitro assay were in following order: cycloheximide < BuOH-fr < Aq-fr < Chl-fr < EtOAc fraction < Hex-fr < MeOH-ext. The cytotoxicity decreased in the following rank order: MeOH-ext > Hex-fr > EtOAc-fr > Chl-fr > Aq-fr > BuOH-fr > cycloheximide. The standard cycloheximide was more cytotoxic to fibroblasts compared to crude extract and its fractions.

### 2.2. Anti-Inflammatory Activity

The anti-inflammatory response of *T. govanianum* rhizomes methanol extract (MeOH-ext) and fractions thereof in carrageenan-induced paw edema model is presented in Table 3 The results indicate that MeOH-ext and its fractions at 50, 100 and 200 mg/kg exhibit significant anti-inflammatory activity comparable to the standard anti-inflammatory drug, diclofenac sodium.

The MeOH-ext and its fractions at doses of 100 and 200 mg/kg showed an anti-inflammatory activity that became significant (*p* < 0.01) in the second phase of inflammation, i.e., 2 h after the injection of carrageenan. The anti-inflammatory effect remained significant in the second phase with a maximum percent inhibitions of 64.6 ± 4.0, 63.5 ± 0.5, 47.5 ± 0.5 and 72.6 ± 3.9 by MeOH-ext, chloroform, ethyl acetate and *n*-butanol fractions respectively. The MeOH-ext and its fractions showed a slightly weaker activity in early phase of inflammation (1–2 h), though *n*-butanol fraction was active at a dose of 100 mg/kg.

In chemiluminescence assay, the in vitro inhibitory effects on the release of reactive oxygen species (ROS) from whole blood by MeOH-ext and its fractions are presented in Table 4. The results show that *n*-butanol fraction exhibit significant inhibition of oxidative burst for whole blood followed by MeOH-ext with IC_50_ ± SD of 16.5 ± 7.5 and 30.8 ± 7.0 µg/mL respectively.

**Figure 1:**
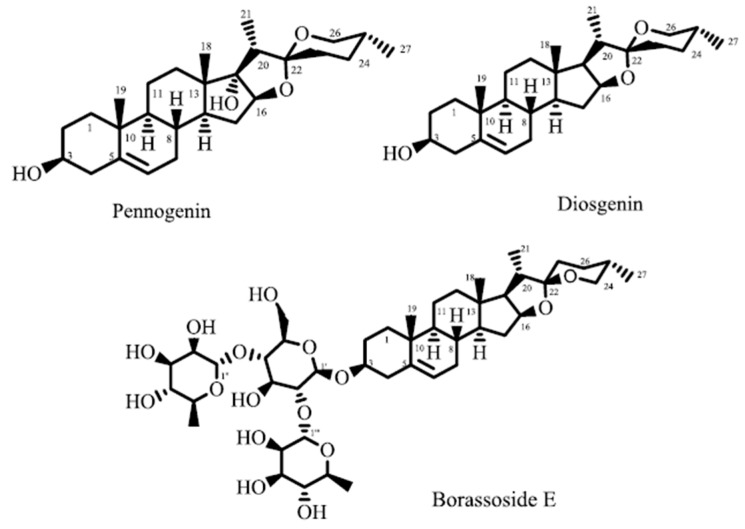
Compounds of *T. govanianum* rhizomes exhibiting oxidative burst inhibitory effect.

Based on these results, isolated compounds i.e., diosgenin, pennogenin and borassoside E (Figure 1) from *T. govanianum* rhizomes were screened for inhibitory effects on release of ROS from whole blood (Table 4). Pennogenin exhibited significant in vitro inhibitory effect by controlling release of ROS with IC_50_ of 5.0 ± 0.8 µg/mL, while borassoside E and diosgenin showed weak inhibition.

Inflammation and reactive oxygen species (ROS) has a relationship of mutual promotion. ROS are associated with inflammatory response and may contribute tissue damages [26]. In addition, oxidative stress is thought to play an important role in the pathogenesis of many chronic inflammatory diseases by different molecular mechanisms [27]. Numerous studies have shown that production of reactive species such as ROS, reactive nitrogen species (RNS) and hypochlorous acid (HOCl) occur at the site of inflammation, thus exacerbating tissue damage as well as the inflammatory cascade [28,29].

Drugs that inhibit the formation or release of these toxic reactive species (or detoxify them) are effective in treating a variety of diseases that involves stimulation of immune cells like AIDS, rheumatoid arthritis and cancer [30]. Luminol-enhanced chemiluminescence assay is based on the detection of intracellular ROS released by opsonized zymosan activated immune cells. A measurement of chemiluminescence is an efficient and highly sensitive method to investigate different kinds of ROS, and is also a suitable method for detection of superoxide anion in biological systems [31].

The carrageenan-induced paw edema model, represent a form of acute inflammation, has been frequently used to assess the anti-inflammatory effects of natural products [32].This is useful model to assess the contribution of mediators involved in vascular changes associated with acute inflammation [33]. Within first hour following carrageenan injection, edema is induced by the release of various mediators such as histamine, bradykinin and 5-HT (5-hydroxytriptamine or serotonin), but not through prostaglandins (PG). These mediators, following activation of their receptors on endothelial cells, trigger iNOS (inducible nitric oxide synthase) activation and the generation of nitric oxide (NO), and RNS. In mice, following the intraplantar injection of carrageenan, tissue necrosis factor as well as cytokines such as IL-1 and IL-2 are produced [34]. Cyclooxygenase-2 (COX-2) is also induced within 2 h post carrageenan administration [35]. The NOS and COX pathways appear to operate together to amplify the inflammatory response. The dual inhibition of NO and PG obtained with NOS inhibitors could account for their marked anti-inflammatory activity [33]. Neutrophil infiltration in response to carrageenan and following membrane NADPH oxidase activation generates an oxygen respiratory burst giving rise to oxygen-derived free radicals and eliciting immune cell recruitment, which, in turn, produces tissue damage [36].

The ability of *T. govanianum* rhizomes MeOH-ext, fractions and isolated compounds thereof to inhibit release of ROS from whole blood after serum opsonized zymosan activation, may be a consequence of either scavenging of ROS during oxidative burst or inhibition of enzymes involved in their production such as NADPH oxidase, superoxide dismutase (SOD), catalases and peroxidases. As there is correlation between in vitro anti oxidative activities and in vivo anti-inflammatory activities [37], thus our in vitro results strongly support significant in vivo anti-inflammatory activity by extract and fractions in the carrageenan-induced paw edema model. Furthermore, diosgenin, a saponin aglycon found in a variety of plants, has anti-inflammatory properties, and it has been reported that diosgenin at dose of 400 µg/kg is responsible for maximum anti-inflammatory effect (82.25%) in carrageenan induced paw edema model [38]. Since, diosgenin is one of the major metabolite of *T. govanianum* rhizomes [23], it confirms our in vitro and in vivo anti-inflammatory results.

### 2.3. Analgesic Activity

#### 2.3.1. Abdominal Constriction Assay (Tonic Visceral Chemically-Induced Nociception)

**Figure 2:**
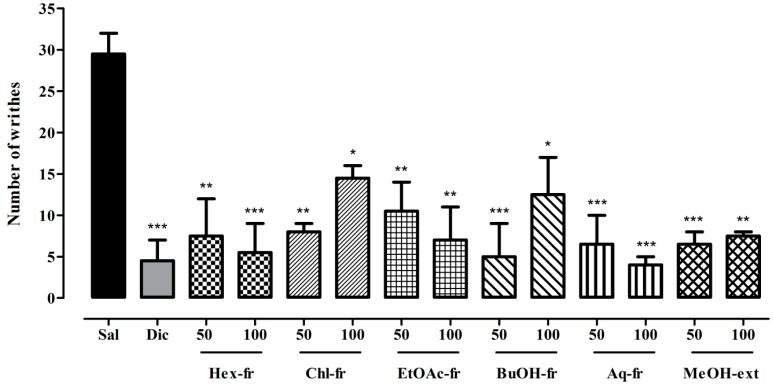
Antinociceptive activity of *Trillium govanianum* rhizome methanol extract and fractions in tonic visceral chemically-induced nociception. Values are expressed as mean ± SEM. ANOVA followed by Dunnett’s *post hoc* test: * *p* < 0.05, ** *p* < 0.01, *** *p* < 0.001 compared to saline treated group; *N* = 6. Dic = diclofenac.

The results of MeOH-ext and its solvent soluble fractions in tonic visceral chemical-induced nociception assay are presented in Figure 2 (Table S1; Supplementary materials), which indicates that 50 and 100 mg/kg doses significantly attenuate acetic acid induced writhes for Hex-fr (*p* < 0.01, *p* < 0.001), Chl-fr (*p* < 0.01, *p* < 0.05), EtOAc-fr (*p* < 0.01), BuOH-fr (*p* < 0.001, *p* < 0.05), Aq-fr (*p* < 0.001), and MeOH-ext (*p* < 0.001, *p* < 0.01), respectively. The antinociceptive activity was comparable to the standard drug diclofenac sodium, which significantly relieved (*p* < 0.001) the tonic visceral chemical-induced nociceptive pain.

Abdominal constriction assay (acetic acid-induced writhing method) is widely used for evaluation of peripheral anti-nociceptive activity [39]. In tonic visceral chemical-induced nociception model, injection of acetic acid into the peritoneal cavity of mice has been attributed to the release of arachidonic acid (AA), which contributes in prostaglandin synthesis through cyclooxygenase enzyme [8]. The special nerve endings that sense pain are very sensitive to prostaglandin. When prostaglandins are released, nerve endings respond to it through prostaglandin E2 receptor, transmitting the pain and injury messages to brain and induce strong contraction tracked by extension of the hind limbs (writhing). This visceral pain model is simple, reliable and rapid for investigation of peripheral analgesics.

#### 2.3.2. Hot Plate Test (Acute Phasic Thermal Nociception)

**Figure 3:**
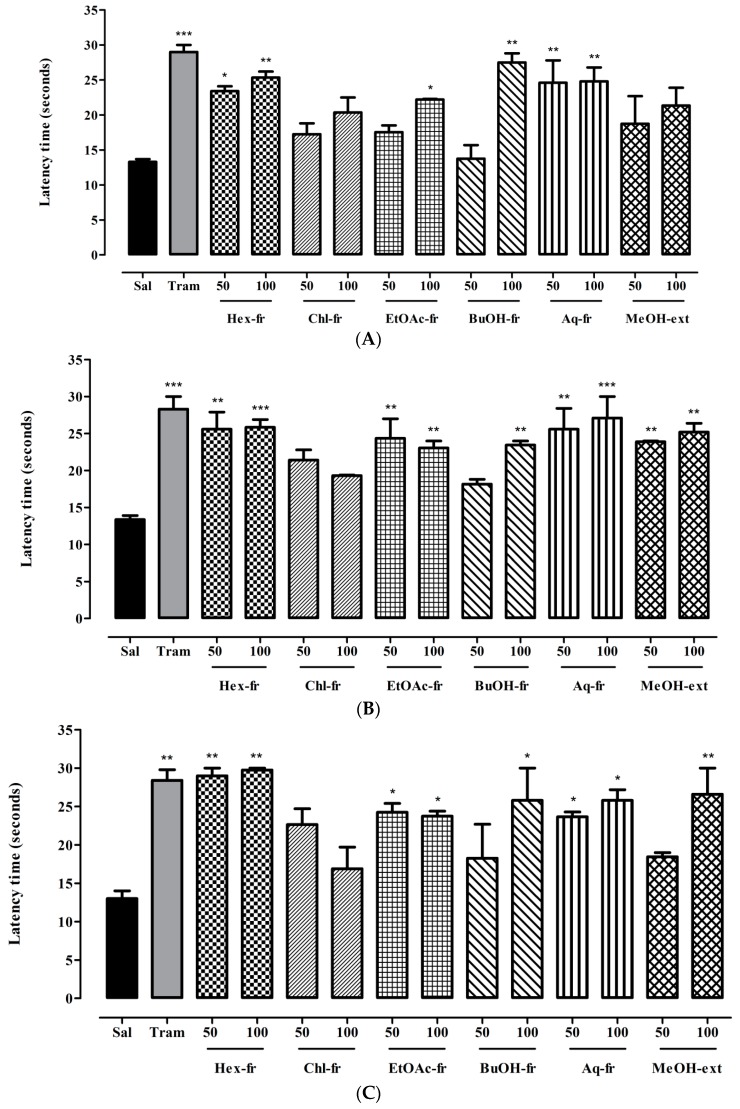

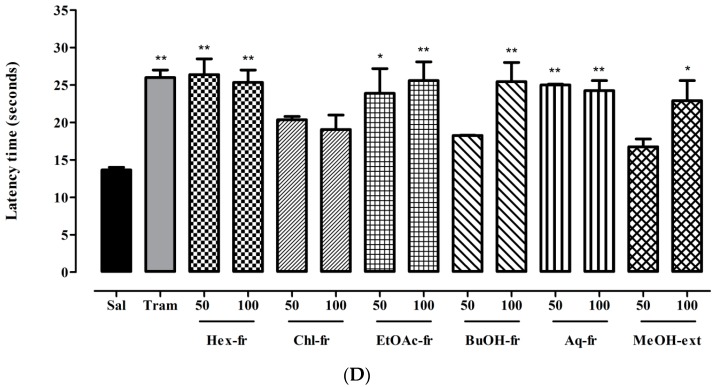
(**A**) Antinociceptive effect of *Trillium govanianum* rhizomes MeOH-extract its fractions (mg/kg) after 30 min. Sal, saline solution; Tram, tramadol; (**B**) Antinociceptive effect of *Trillium govanianum* rhizomes MeOH-extract its fractions (mg/kg) after 60 min. Sal, saline solution; Tram, tramadol; (**C**) Antinociceptive effect of *Trillium govanianum* rhizomes MeOH-extract its fractions (mg/kg) after 90 min. Sal, saline solution; Tram, tramadol; (**D**) Antinociceptive effect of *Trillium govanianum* rhizomes MeOH-extract its fractions (mg/kg) after 120 min. Sal, saline solution; Tram, tramadol. ANOVA followed by Dunnett’s *post hoc* test: * *p* < 0.05, ** *p* < 0.01, *** *p* < 0.001 compared to saline treated group; *N* = 6.

The MeOH-ext and its fractions were evaluated for analgesic effect in the acute phasic thermal nociceptive pain. The results shown in Figure 3A–D (Table S2; Supplementary materials) indicate that, after 30 min, compared to normal saline, significant attenuation of thermal-induced nociception was observed for Hex-fr at 50 mg/kg (*p* < 0.05) and 100 mg/kg (*p* < 0.01), EtOAc-fr at 100 mg/kg (*p* < 0.05), BuOH-fr at 100 mg/kg (*p* < 0.01), and Aq-fr at 50 and 100 mg/kg (*p* < 0.01) (Figure 3A). After 60 min, significant analgesic effect was observed for Hex-fr at 50 mg/kg (*p* < 0.01) and 100 mg/kg (*p* < 0.001), EtOAc-fr at 50 and 100 mg/kg (*p* < 0.01), BuOH-fr at 100 mg/kg (*p* < 0.01), Aq-fr at 50 mg/kg (*p* < 0.01) and 100 mg/kg (*p* < 0.001), and MeOH-ext at both doses (*p* < 0.01) (Figure 3B). Likewise, significant protection against thermal-induced nociception after 90 min was observed with all test doses of Hex-fr (*p* < 0.01), EtOAc-fr (*p* < 0.05), Aq-fr (*p* < 0.05) as well as with 100 mg/kg dose of BuOH-fr (*p* < 0.05) and MeOH-ext (*p* < 0.01) (Figure 3C). Moreover, analgesic effect produced after 120 min was significant for all test doses of Hex-fr (*p* < 0.01), EtOAc-fr (*p* < 0.05, *p* < 0.01) and Aq-fr (*p* < 0.01), and for only 100 mg/kg doses of BuOH-fr (*p* < 0.01) and MeOH-ext (*p* < 0.05) (Figure 3D).

Hot plate test is one of the most common tests employed in evaluation of central analgesic potential of drugs. The mice paws are very sensitive to heat at temperatures, which are not damaging to skin. Mice show escape responses to the nocifensive stimulus and are manifested as jumping, licking or withdrawal of the paws. These responses take prolonged time to appear after administration of centrally acting analgesic drugs. Thus, the hot plate test model measures different responses to acute nociceptive or non-inflammatory inputs and is one of the models normally used for studying central antinociceptive activity [40].

It has been reported that steroidal saponins are among the major chemical constituents in medicinal preparations responsible for most of the anti-inflammatory and analgesic activities. Recent reports also indicate that saponins suppress the expression of iNOS and COX-2, thus resulting in a noticeable lowering of prostaglandin E_2_ levels [41,42]. The phytochemical analysis of *T. govanianum* rhizomes showed that it is a rich source of steroids and saponins (Figure 1) [36], therefore it is assumed that the observed effects are due to the presence of these metabolites in *T. govanianum* [43,44].

It is worth mentioning that mice were selected as the species of choice in these specific tests because they are noticeably sensitive not only to opioid-mediated effects, but also to coexistent non-steroidal anti-inflammatory drugs (NSAIDs) activity [45,46]. Moreover, the murine abdominal constriction assays as well as the hot plate test detect dose-dependent anti-nociception in this species quite well [47,48].

## 3. Experimental Section

### 3.1. Plant Material

*T. govanianum* rhizomes were collected from Khyber Pakhtunkhwa, Upper Dir, Kohistan valley (34°54′ and35° 52′ North latitudes, 72°43′ and 73°57′ East longitudes), in August, 2013. The plant was authenticated by Mr. Ghulam Jelani (Curator) of the Department of Botany, University of Peshawar. Afterwards, a voucher specimen [Bot. 20092 (PUP)] of this plant was deposited in the herbarium for record.

### 3.2. Extraction and Fractionation

The shade-dried rhizomes of *T. govanianum* (7 kg) were grounded and extracted with MeOH (40 L) at room temperature, three times for seven days (3 × 40 L). The combined methanolic extract was evaporated to dryness that yielded a brownish gummy residue (512 g). It was further fractionated (solid-liquid partition) into hexane (Hex-fr, 81 g), chloroform (Chl-fr, 94 g), ethyl acetate (EtOAc-fr, 85 g), *n*-butanol (BuOH-fr, 115 g), and aqueous (Aq-fr, 205 g) fractions. The chloroform and *n*-butanol fractions were subjected to column chromatography and elution was carried out with mixture of hexane, chloroform, EtOAc and MeOH in increasing order of polarity. The compounds diosgenin and pennogenin were obtained at 20 and 25% EtOAc/hexane solvent system, respectively, while borassoside was obtained at 5% MeOH/EtOAc solvent system. The doses of the extract and its fractions used in the in vivo assays were selected in relation to the usual therapeutic doses of the respective standard drugs (controls) utilized in the pharmacological activities.

### 3.3. Animals

BALB/c mice of either sex (21–35 g) were maintained and acclimatized at 25 ± 2 °C under a 12 h dark/light cycle. Food and water were provided *ad libitum*. The experimental procedures were approved by the Ethical Committee of the Department of Pharmacy, University of Peshawar, Pakistan (14/EC/Pharm).

### 3.4. Acute Toxicity Test

#### 3.4.1. In Vivo Toxicity

The acute toxicity test was performed according to the organization for economic co-operation for development (OECD) guidelines for the testing of chemicals, Test No. 423 (OECD guidelines, 2001) and the same methodology is also used for screening of medicinal plants [49]. The animals were divided into six groups each consisting of six mice (*n* = 6). *T. govanianum* extract was administered in doses of 250, 500, 1000, 1500, 3000 and 6000 mg/kg body weight through an oral gavage tube and the morbidity was observed continuously for the first 2 h and mortality up to 24 h post dose administration [50]. The animals were observed for spontaneous activity, aggressiveness, cyanosis, ataxia, tail pinch response, righting reflex, writhing, convulsions, catalepsy and bizarre behavior. The 50% mortality among the animals indicated the toxicity concentration of the extract which was calculated using probit analysis.

#### 3.4.2. In Vitro Toxicity

The in vitro cytotoxic activity of methanol extract and fractions thereof was determined by following MTT assay, with slight modification [51,52], on 3T3 cell lines.

### 3.5. Anti-Inflammatory Assay

#### 3.5.1. Carrageenan Induced Paw Edema Assay

The anti-inflammatory activity was performed using the carrageenan induced paw edema model in mice [53]. Briefly, BALB/c mice (25–30 g) were randomly divided into five groups (*n* = 6). Group I was treated with normal saline (10 mL/Kg), group II with diclofenac sodium (10 mg/Kg), while the other groups were treated with *T. govanianum* rhizomes MeOH-ext and its fractions (50, 100 and 200 mg/Kg; *p.o.*). After 30 min of drug treatment, 0.05 mL low molecular weight carrageenan (MW 20–30 kD, 1% *w*/*v* in 0.9% NaCl *w*/*v* solution) was injected subcutaneously in the sub plantar tissue of the right hind paw of each mouse. The inflammation was measured using a digital plethysmometer (LE 7500 plan lab S.L) immediately after injection of carrageenan and, then, at 1, 2, 3, 4 and 5 h intervals. The average paw swelling in tested samples as well as standard treated animals was compared with that of control and percent inhibition (anti-inflammatory activity) of edema was determined using the following formula:

Percentage of edema inhibition (%) = (VA − VB/VA) × 100

where VA is the edema volume of control and VB is the volume of paw edema in treated group.

#### 3.5.2. Chemiluminescence Assay

Luminol-enhanced chemiluminescence assay was performed, as per previously reported method [54]. In brief, 25 µL of diluted whole blood HBSS^++^ (Hanks Balanced Salt Solution, containing calcium chloride and magnesium chloride) (Sigma, St. Louis, MO, USA) was incubated with 25 µL of three different concentrations of test compounds (1, 10 and 100 µg/mL), each in triplicate. Control wells received HBSS^++^ and cells, but no compound. Test was performed in white half area 96 well plates (Costar, New York, NY, USA), which was incubated at 37 °C for 15 min in the thermostat chamber of luminometer (Labsystems, Helsinki, Finland). After incubation, 25 µL of serum opsonized zymosan (SOZ) (Fluka, Buchs, Switzerland) and 25 µL of intracellular reactive oxygen species detecting probe, luminol (Research Organics, Cleveland, OH, USA), were added into each well, except blank wells (containing only HBSS^++^). The level of ROS was recorded by a luminometer and expressed as relative light units (RLU). Drug ibuprofen was used as positive control.

### 3.6. Analgesic Activity

#### 3.6.1. Abdominal Constriction Assay

The acetic acid induced abdominal constriction assay was performed to determine the peripheral antinociceptive effect [47]. The animals (mice) were withdrawn from food 2 h before the start of experiment. *T. govanianum* rhizomes extract and fractions thereof were administered orally through an oral gavage tube at doses of 50 and 100 mg/kg. Diclofenac sodium was used as standard and was administered at a dose of 50 mg/kg, (*i.p.*). After 1 h of test drug and standard drug treatments, all animals were injected with 1% acetic acid, (*i.p.*). The numbers of writhes were counted after 5 min of acetic acid injection up to 20 min.

#### 3.6.2. Hot Plate Test

The central analgesic effect of *T. govanianum* rhizomes was evaluated by using hot plate test method [55]. Animals (mice) were withdrawn from food 2 h prior to experiment. All animals were screened for pre-test latency time and only those animals having a pre-test latency time of <15 s were selected for experiment. A cut off time, 30 s was set to avoid any thermal injury. *T. govanianum* rhizomes extract and its fractions were administered orally through an oral gavage tube at doses of 50 and 100 mg/kg. Tramadol served as positive control and was administered at a dose of 30 mg/kg, (*i.p.*). After 1 h of extract/fractions and 30 min of standard drug administration, the latency time was measured at 30, 60, 90 and 120 min using a hot plate (Harvard apparatus, Kent, UK) maintained at 54 ± 0.1 °C.

### 3.7. Statistical Analysis

Animals were randomly assigned to each treatment groups. Results are presented as mean ± SD or SEM. Statistical comparisons were carried out by one way ANOVA followed by Dunnett’s, *post-hoc* test using GraphPad Prism 5 (GraphPad Software Inc., San Diego, CA, USA). The LD_50_ value in the acute toxicity test was determined by Probit analysis using Minitab version 17.1.0 (Minitab Inc., State College, PA, USA). A *p* value of 0.05 was considered as significant.

## 4. Conclusions

The traditional medicinal use of *T. govanianum* rhizomes to treat pain and inflammation can be validated by findings in our study, where the extract, its fractions and isolated compounds afforded selective antinociceptive and anti-inflammatory properties in animal models. Therefore, *T. govanianum* rhizomes may serve as a potential source of novel compounds effective in pain and inflammatory conditions.

## Figures and Tables

**Table 1:** In vivo toxicity test of *Trillium govanianum* rhizome methanol extract.

Dose (mg/kg)	Total Number of Mice = 6		Percent Lethality	LD_50_ (mg/kg)
	No. of Dead Mice	No. of Survived Mice		
150	0	6	0	2030.4 (1488.8–3069.0) *
500	0	6	0	
1000	1	5	16	
1500	2	4	33	
3000	5	1	83	
6000	6	0	100	

* 95% confidence limit in parentheses.

**Table 2:** In vitro cytotoxicity assay of *Trillium govanianum* rhizome methanol extract and its fractions on NIH 3T3 mouse embryo fibroblasts.

Sample	IC_50_ (µg/mL)
MeOH-ext	7.89 ± 0.43
Hex-fr	5.78 ± 0.34
Chl-fr	3.69 ± 0.77
EtOAc-fr	3.95 ± 0.25
BuOH-fr	2.59 ± 0.14
Aq-fr	3.07 ± 0.29
Cycloheximide	0.73 ± 0.12

Values are expressed as mean ± SD of three separate experiments.

**Table 3:** Anti-inflammatory activity of *Trillium govanianum* rhizome methanol extract and fractions in carrageenan-induced paw edema assay.

Sample	Dose (mg/kg)	1st h	2nd h	3rd h	4th h	5th h
Diclofenac sodium	10	27.3 ± 2.7 ***	47.6 ± 0.8 ***	67.0 ± 2.5 ***	70.6 ± 0.6 ***	74.3 ± 0.6 ***
MeOH-ext	50	8.0 ± 1.7 *	18.0 ± 1.7 **	22.6 ± 2.3 ***	32.0 ± 5.2 ***	34.0 ± 5.2 ***
	100	12.0 ± 1.1 **	44.3 ± 4.6 ***	65.0 ± 4.6 ***	63.6 ± 1.7 ***	66.3 + 4.6 ***
	200	19.0 ± 2.3 *	44.6 ± 3.8 ***	62.6 ± 3.7 ***	62.6 ± 1.4 ***	64.6 ± 4.0 ***
Chl-fr	25	4.0 ± 1.0 *	8.5 ± 0.5 *	21.3 ± 3.5 **	35.0 ± 1.0 ***	42.5 ± 2.5 ***
	50	4.0 ± 2.0 **	18.8 ± 1.0 **	20.3 ± 2.0 **	43.0 ± 1.0 ***	58.0 ± 1.0 ***
	100	12.1 ± 3.0 *	21.5 ± 0.5 **	43.5 ± 3.5 ***	45.0 ± 1.0 ***	63.5 ± 0.5 ***
EtOAc-fr	25	3.5 ± 0.5 *	9.5 ± 0.5 *	18.0 ± 3.0 **	18.5 ± 1.5 **	39.5 ± 0.5 ***
	50	10.5 ± 0.5 *	16.0 ± 1.0 **	29.5 ± 0.5 ***	30.5 ± 2.5 ***	44.5 ± 3.5 ***
	100	9.5 ± 0.5 *	22.5 ± 1.5 **	30.5 ± 0.5 ***	33.0 ± 2.0 ***	47.5 ± 0.5 ***
BuOH-fr	25	12.9 ± 3.7 *	14.3 ± 5.5 *	35.2 ± 1.0 ***	58.0 ± 1.5 ***	61.0 ± 3.4 ***
	50	18.8 ± 3.4 ***	46.6 ± 4.9 ***	68.6 ± 2.3 ***	66.0 ± 1.7 ***	69.0 ± 4.3 ***
	100	25.6 ± 1.7 ***	47.6 ± 2.1 ***	68.6 ± 1.4 ***	70.3 ± 2.6 ***	72.6 ± 3.9 ***

Percent inhibition of paw edema is expressed as mean ± SEM. One-way ANOVA followed by Dunnett’s *post hoc* test: * *p* < 0.05, ** *p* < 0.0, *** *p* < 0.001 compared to saline control; *N* = 6 mice per group.

**Table 4:** Inhibitory effect on release of ROS by *Trillium govanianum* methanol extract, fractions and isolated compounds.

Samples	IC_50_ ± SD (µg/mL)
MeOH-ext	30.8 ± 7.0
Hex-fr	107.1 ± 38.4
Chl-fr	81.6 ± 24.6
EtOAc-fr	114.8 ± 2.3
BuOH-fr	16.5 ± 7.5
Pennogenin	5.0 ± 0.8
Borassoside E	31.5 ± 6.6
Diosgenin	53.2 ± 2.7
Ibuprofen (Positive control)	11.2 ± 1.9

Values represent mean ± SEM of three independent experiments.

## Supplementary Materials

The supplementary materials are available online at: http://www.mdpi.com/1420-3049/21/8/1095/s1.

## Abbreviations

MeOH-ext	methanol extract
Chl-fr	chloroform fraction
EtOAc-fr	ethyl acetate fraction
BuOH-fr	*n*-butanol fraction
Hex-fr	*n*-hexane fraction
Aq-fr	aqueous fraction
COX-2	cyclooxygenase-2
HBSS^++^	Hanks balanced salt solution
iNOS	inducible nitric oxide synthase
RLU	relative light units
ROS	reactive oxygen species
SOZ	serum opsonized zymosan
PG	prostaglandins
NO	nitric oxide
HOCl	hypochlorous acid
SOD	superoxide dismutase
IL	interleukin
