# Supplementary material for: Beneficial Effects of Trillium govanianum Rhizomes in Pain and Inflammation
Source: Molecules. 2016 Aug 20;21(8):1095. doi: 10.3390/molecules21081095 (PMC6274187; doi:10.3390/molecules21081095)
Supplement: Supplementary file 1 [file molecules-21-01095-s001.pdf]

Supplementary Materials: Beneficial Effects of *Trillium govanianum* Rhizomes in Pain and Inflammation

Shafiq Ur Rahman, Achyut Adhikari, Muhammad Ismail, Muhammad Raza Shah, Muhammad Khurram, Muhammad Shahid, Farman Ali, Abdul Haseeb, Fazal Akbar and Marcello Iriti

Table S1. Antinociceptive effect of *T. govanianum* rhizome MeOH-ext and its fractions in tonic-visceral chemically-induced nociception.

Sample	Dose (mg/kg)	Number of Writhes
Saline	10 mL/kg	29.50 ± 2.50
Diclofenac	50	4.50 ± 2.50 ***
n-Hex-fr	50	7.50 ± 4.50 **
	100	5.50 ± 3.50 ***
CHL-fr	50	8.00 ± 1.00 **
	100	14.50 ± 1.50 *
EtOAc-fr	50	10.50 ± 3.50 **
	100	7.00 ± 4.00 **
BuOH-fr	50	5.00 ± 4.00 ***
	100	12.50 ± 4.50 *
Aq-fr	50	6.50 ± 3.50 ***
	100	4.00 ± 1.00 ***
MeOH-Ext	50	6.50 ± 1.50 ***
	100	7.50 ± 0.50 **

Values expressed as mean ± SEM. ANOVA followed by Dunnett's post hoc test. * $p < 0.05$, ** $p < 0.01$, *** $p < 0.001$ compared to saline treated group, $n = 6$ mice per group.

Table S2. Antinociceptive effect of MeOH-ext of *T. govanianum* rhizome and its fractions in thermal induced nociception.

Sample	Dose (mg/kg)	30 min	60 min	90 min	120 min
Saline	10 mL/kg	13.30 ± 0.40	13.35 ± 0.55	13.00 ± 1.00	13.65 ± 0.35
Tramadol	30	29.00 ± 1.00 ***	28.30 ± 1.70 ***	28.40 ± 1.40 *	26.00 ± 1.00 **
Hex-fr	50	23.40 ± 0.70 *	25.60 ± 2.30 **	29.00 ± 1.00 **	26.40 ± 2.10 **
	100	25.35 ± 0.85 **	25.85 ± 1.05 ***	29.75 ± 0.25 **	25.35 ± 1.65 **
Chl-fr	50	17.25 ± 1.55	21.40 ± 1.40 *	22.65 ± 2.05 *	20.35 ± 0.45 *
	100	20.35 ± 2.15 *	19.30 ± 0.10	16.90 ± 2.80	19.05 ± 1.95
EtOAc-fr	50	17.55 ± 0.95	24.35 ± 2.65 **	24.25 ± 1.15 *	23.90 ± 3.30 *
	100	22.20 ± 0.10 *	23.05 ± 0.95 **	23.75 ± 0.65 *	25.60 ± 2.50 **
BuOH-fr	50	13.75 ± 1.95	18.15 ± 0.65	18.25 ± 4.45	18.25 ± 0.05
	100	27.50 ± 1.30 **	23.45 ± 0.55 **	25.80 ± 4.20 *	25.45 ± 2.55 **
Aq-fr	50	24.60 ± 3.20 **	25.60 ± 2.80 **	23.65 ± 0.65 *	25.00 ± 0.10 **
	100	24.80 ± 2.00 **	27.10 ± 2.90 ***	25.80 ± 1.40 *	24.25 ± 1.35 **
MeOH-Ext	50	18.75 ± 3.95 *	23.90 ± 0.10 **	18.45 ± 0.55	16.75 ± 1.05
	100	21.35 ± 2.55 *	25.20 ± 1.20 **	26.60 ± 3.40 **	22.90 ± 2.70 *

Values expressed as mean ± SEM. ANOVA followed by Dunnett's post hoc test. * $p < 0.05$, ** $p < 0.01$, *** $p < 0.001$ compared to saline treated group, $n = 6$.
